# Small organic molecules containing amorphous calcium phosphate: synthesis, characterization and transformation

**DOI:** 10.3389/fbioe.2023.1329752

**Published:** 2024-01-12

**Authors:** Abhishek Indurkar, Pawan Kudale, Vitālijs Rjabovs, Ivo Heinmaa, Öznur Demir, Matvejs Kirejevs, Kristaps Rubenis, Ganesh Chaturbhuj, Māris Turks, Janis Locs

**Affiliations:** ^1^ Rudolfs Cimdins Riga Biomaterials Innovations and Development Centre of RTU, Institute of General Chemical Engineering, Faculty of Materials Science and Applied Chemistry, Riga Technical University, Riga, Latvia; ^2^ Baltic Biomaterials Centre of Excellence, Headquarters at Riga Technical University, Riga, Latvia; ^3^ Department of Pharmaceutical Sciences and Technology, Institute of Chemical Technology, Mumbai, India; ^4^ Institute of Technology of Organic Chemistry, Faculty of Materials Science and Applied Chemistry, Riga Technical University, Riga, Latvia; ^5^ National Institute of Chemical Physics and Biophysics, Tallinn, Estonia

**Keywords:** amorphous calcium phosphate, organic compounds, ascorbate, itaconate, glutamate, biomaterials, bone tissue engineering, biomimetics

## Abstract

As the primary solid phase, amorphous calcium phosphate (ACP) is a pivotal precursor in cellular biomineralization. The intrinsic interplay between ACP and Howard factor underscores the significance of understanding their association for advancing biomimetic ACP development. While organic compounds play established roles in biomineralization, this study presents the synthesis of ACP with naturally occurring organic compounds (ascorbate, glutamate, and itaconate) ubiquitously found in mitochondria and vital for bone remodeling and healing. The developed ACP with organic compounds was meticulously characterized using XRD, FTIR, and solid-state ^13^C and ^31^P NMR. The morphological analysis revealed the characteristic spherical morphology with particle size close to 20 nm of all synthesized ACP variants. Notably, the type of organic compound strongly influences true density, specific surface area, particle size, and transformation. The *in vitro* analysis was performed with MC3T3-E1 cells, indicating the highest cell viability with ACP_ASC (ascorbate), followed by ACP_ITA (itaconate). The lowest cell viability was observed with 10 %w/v of ACP_GLU (glutamate); however, 1 %w/v of ACP_GLU was cytocompatible. Further, the effect of small organic molecules on the transformation of ACP to low crystalline apatite (Ap) was examined in Milli-Q^®^ water, PBS, and α-MEM.

## 1 Introduction

Amorphous calcium phosphate (ACP) has attracted much attention since it is the first calcium phosphate (CaP) phase synthesized and stabilized by cells and acts as a precursor of hydroxyapatite (HAp) (Combes and Rey, 2010). ACP provides a reservoir of calcium and phosphate ions that can be utilized for bone growth and regeneration. It is formed in the early stages of mineralization, which gradually crystallizes to HAp (Barrère et al., 2006). Naturally, ACP is stabilized by an organic compound known as the “Howard factor,” whose exact chemical properties are unknown (Howard and Thomas, 1968; Lehninger, 1970). The association of Howard factor with ACP occurs in mitochondria, indicating that ACP forms a complex natural composite of inorganic CaP associated with an organic compound. In literature, synthetic ACP was prepared via numerous routes; however, the biogenic organic additives were not considered (Combes and Rey, 2010). The prerequisite for developing biomimetic ACP is the association of organic compounds present in mitochondria.

Considering this, numerous attempts have been undertaken in the development of ACP composite materials using macromolecules such as osteopontin, osteocalcin, dentin matrix protein, bone sialoprotein, dentin phosphoprotein, matrix extracellular protein, connexin 43, casein phospho-peptide, α_2_HS-glycoproteins, fibrin, and albumin (Reynolds, 1998; Makowski and Ramsby, 2001; Gajjeraman et al., 2007; Yang et al., 2010; Yarbrough et al., 2010; Syed-Picard et al., 2013; Padovano et al., 2015; Zhao et al., 2018; Iline-Vul et al., 2020; Erceg and Dutour Sikirić, 2022; Nakamura et al., 2022; Indurkar et al., 2023a). However, *Becher et al.* have identified that many biological ubiquitous small organic molecules can inhibit the conversion of ACP to HAp at their respective tissue concentration (Becker, 1977). Considering these findings, ACP composites were developed using small organic molecules of polycarboxylate (such as citrate, succinate, acetate, and several amino acids), pyrophosphate, phosphocitric acid, polyphosphates as well as di-and-triphosphate nucleotide (Ikawa et al., 2009; Chinopoulos and Adam-Vizi, 2010; Grover et al., 2013; Stipniece et al., 2016; Feng et al., 2020; Indurkar et al., 2023b).

**FIGURE 1:**
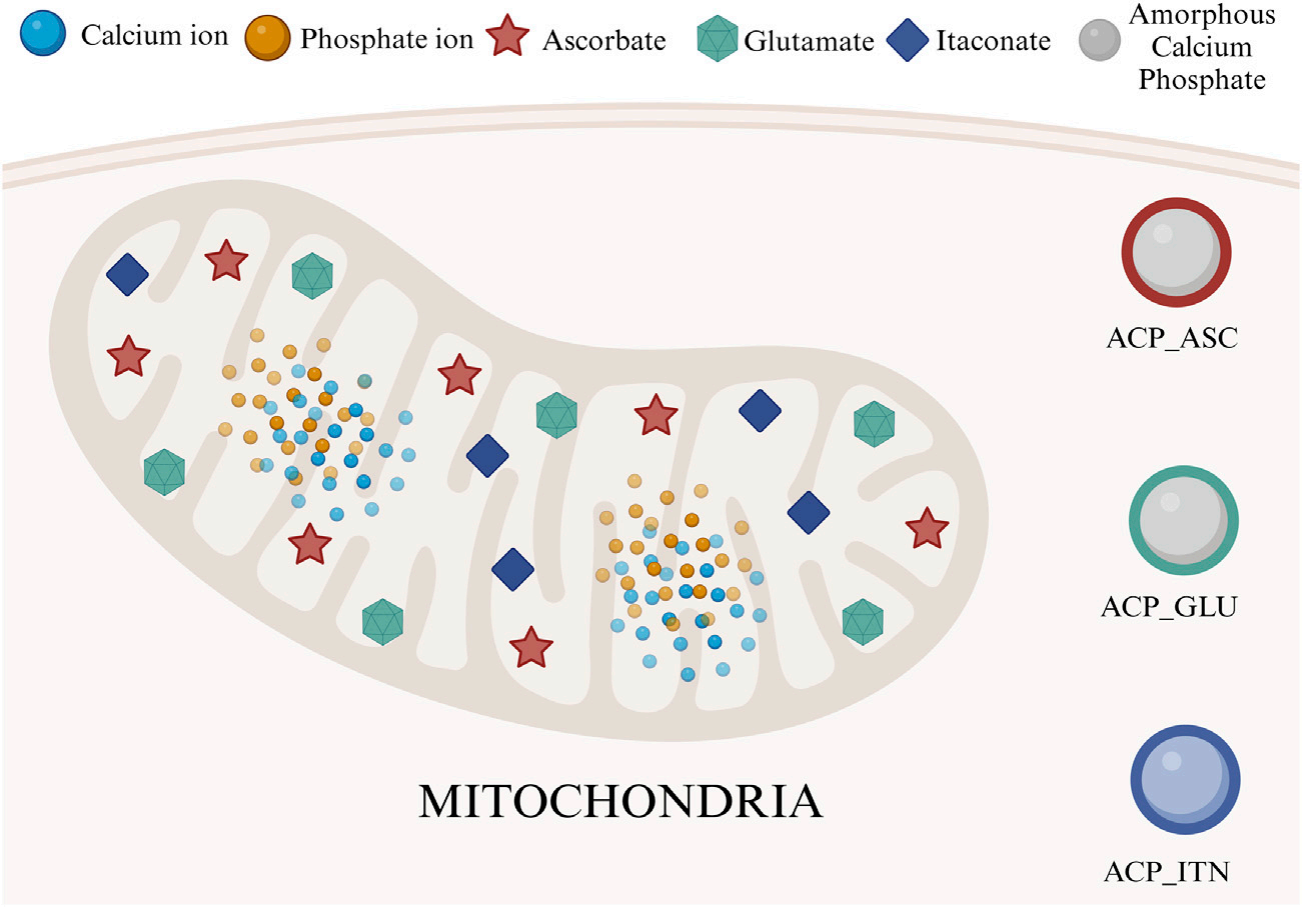
Mitochondria is an epicenter of various small organic compounds and ACP synthesis. In this study, we have utilized ascorbate, glutamate, and itaconate to develop composite ACP nanoparticles.

Mitochondria, which is the epicenter of numerous biochemical cycles, result in the formation of many small organic molecules (Scheffler, 2002; Osellame et al., 2012). Considering that ACP was associated with organic compounds in mitochondria, we have screened molecules based on their functions in bone remodeling and regeneration, such as ascorbate, glutamate, and itaconate, as shown in Figure 1.

Ascorbate (ascorbic acid or vitamin C) is crucial in collagen synthesis and is a vital organic compound in connective tissues and bone (Murad et al., 1981). Collagen provides structure and flexibility to the bone, enabling it to withstand mechanical stress. Without sufficient ascorbate, collagen synthesis is impaired, weakening the bone structure and increasing susceptibility to bone fractures (Gunson et al., 2013). Furthermore, osteogenic cell differentiation depends on ascorbate (Aghajanian et al., 2015; Thaler et al., 2022). Despite human’s incapability of ascorbate synthesis, a specific mitochondrial uptake mechanism obtains vitamins from the diet. The vitamin carrier dehydroascorbic acid enters the mitochondria, where it is reduced and accumulated as ascorbic acid (Chen et al., 2022). Human studies have shown a positive relationship between ascorbic acid and biomineralization, emphasizing its importance in maintaining bone health (Chin and Ima-Nirwana, 2018).

Glutamate is an amino acid that plays a significant role in bone remodeling and healing processes. The glutamate receptors are expressed on the osteoblasts, osteoclasts, and bone marrow cells. Activation of glutamate receptors controls the phenotype of osteoblasts and osteoclasts *in vitro* and bone mass *in vivo* (Brakspear and Mason, 2012). Moreover, glutamate has attained the nitrogen balance in a fractured bone, thus accelerating the bone healing process (Polat et al., 2007).

Itaconate is a metabolite that has gained much attention in recent years due to its role in immune regulation and inhibiting the production of proinflammatory molecules (Wang et al., 2022). Inflammation occurs in various bone disorders, such as rheumatoid arthritis and osteoporosis (Shi et al., 2022). Itaconate controls the immune response, thus indirectly controlling bone health. It also affects the function of macrophages, impacting the bone remodeling process (Peace and O'Neill, 2022). Itaconate interferes with the Krebs cycle, and changes in metabolism influences cell function and overall bone health (Yang et al., 2020).

Mitochondrial molecules such as acetate, citrate, ascorbate, glutamate, and itaconate play a major role in bone regeneration and are linked closely to mitochondrial function. As mitochondria are abundant in bone cells, the dysfunction of these organelles can lead to bone-related disorders (Kalani et al., 2014). The novel direction of incorporation of mitochondrial organic molecules in ACP offers a unique strategy to address bone-related disorders, potentially leading to innovative therapeutic interventions. Previously, we have developed ACP and its composite with citrate (ACP_CIT) and acetate (ACP_ACE) (Indurkar et al., 2023b). In this study, the ACP was synthesized with ascorbate (ACP_ASC), glutamate (ACP_GLU), and itaconate (ACP_ITN), were thoroughly characterized, and their cytocompatibility was evaluated. Additionally, transformation rate of ACP containing small organic molecules in different media were assessed.

The mechanism behind the transformation of ACP to low crystalline apatite (Ap) is a subject of ongoing debate. Several proposed mechanisms include dissolution reprecipitation, cluster reorganization, and solution-mediated solid-solid transformation. It is plausible that multiple processes may coincide during transformation (Jin et al., 2021). Previous studies have explored the transformation of ACP in aqueous solutions and have revealed the influence of factors such as pH, temperature, presence of foreign ions, and additives (polyelectrolyte, phospholipids, polyglycols, proteins, etc.), all of which can affect the transformation rate of ACP (Chatzipanagis et al., 2016). In this context, the role of small organic molecules containing ACP has received relatively less attention, with only a few reports available in the literature (Tsuji et al., 2008; Ikawa et al., 2009; Chatzipanagis et al., 2016; Sun et al., 2020).

In this study, we have also explored the transformation of ACP containing small organic molecules (acetate, glutamate, itaconate, ascorbate, and citrate) in three different mediums: Milli-Q^®^ water, phosphate buffer saline (PBS), and alpha-modified minimum essential eagle medium with 10% fetal bovine serum (FBS) and 1% penicillin and streptomycin (α-MEM). The transformation of ACP was studied using X-ray diffraction (XRD) and Fourier-transformed infrared spectroscopy (FTIR) analysis performed at various time points. The results uncover how incorporation of small organic molecules into the ACP affects its crystallization rate in different media.

## 2 Material and methods

Calcium chloride (CAS 10043-52-4), trisodium phosphate (CAS 7601-54-9), sodium hydroxide (CAS 1301-73-2), and itaconic anhydride (CAS 2170-03-8) were procured from Sigma Aldrich, Germany. Calcium glutamate (CAS 19238-49-4) was obtained from BenchChem, United States, and ascorbic acid (CAS 50-81-7) was procured from Enola, Latvia.

### 2.1 Synthesis

To synthesize ACP_GLU, 150 mM calcium glutamate solution was prepared in Milli-Q^®^ water. For the synthesis of ACP_ITN and ACP_ASC, a similar procedure was followed, starting with the preparation of 150 mM calcium chloride was prepared in Milli-Q^®^ water, followed by the addition of 150 mM itaconic acid (ACP_ITN) and ascorbic acid (ACP_ASC) respectively. Afterwards, the pH of the calcium precursor solution was carefully adjusted to 11.5 using 3 M NaOH solution.

Following the pH adjustment, an equal amount (150 mL) of 100 mM of trisodium phosphate solution was added rapidly to the respective calcium salt solution (total volume 300 mL). Throughout the process, continuous stirring was maintained. Immediately after precipitation, the suspension underwent centrifugation at 3,000 rpm for 5 min, and the resulting precipitate was washed thrice with Milli-Q^®^ water. Later, the centrifuge tube containing the precipitate was immersed in liquid nitrogen for 15 min, followed by freeze-drying for 72 h. The obtained power was stored in airtight containers until further characterization. Similarly, pure ACP, ACP_ACE and ACP_CIT were synthesized and characterized previously (Indurkar et al., 2023b).

### 2.2 Characterization

The phase composition of synthesized ACP variants were determined using X-ray diffraction, performed with a PANalytical Aeris diffractometer (Netherlands). The diffraction data were collected at 40 kV and 15 mA in a step mode with a step size of 0.04°, in the 2θ range from 10° to 60°.

The Fourier-transformed infrared spectroscopy (FTIR) analysis was performed using a Nicolet iS50 FT-IR spectrometer (Thermo Scientific, Waltham, MA, United States). Experiments were performed in transmission mode from the wavenumber ranging from 4,000 to 400 cm^−1^ with a resolution of 4 cm^−1^ (64 scans).

Solid-state CP MAS ^13^C NMR spectra were recorded on Bruker AVANCE-II spectrometer at 14.1 T magnetic field using a home-built double resonance magic-angle-spinning probe for 4 x 25 mm Si_3_N_4_ rotors. The spinning speed of the sample was 12.5 kHz, the duration of the ramped polarization transfer pulse was 1 ms, and the relaxation delay between accumulations was 5 s. From 15,000 to 32,000, scans were accumulated for the spectra. The intensities were normalized to the number of scans and the sample’s weight. Solid-state ^31^P NMR spectra were recorded on JOEL, ECZR 600 NMR spectrometer. The experiment was performed with a 90° single pulse at a MAS frequency of 10 Hz with 2048 scans and a relaxation delay of 3 s.

The morphology and particle size of synthesized ACPs were evaluated by FEG-TEM (Tecnai G2 F30, United States) operated at 300 kV. The sample preparation was as follows: a small amount of powder was dispersed in isopropyl alcohol and sonicated in an ultrasonic bath. Further, the samples were placed on a carbon-coated grid and dried before analysis.

The true density of ACPs was analyzed by a helium pycnometer Micro UltraPyc 1200e (Quantachrome instruments, Boynton, FL, United States). Initially, the calibration was performed using a stainless-steel calibration sphere. It was followed by adding a known amount of ACP powder into the sample holder and purging it with helium gas in pulse mode (30 pulses). Subsequently, the sample volume was analyzed by pressurizing it at 10 psi with helium gas. The true density was calculated using sample weight and the analyzed sample volume. The analysis of each ACP was performed in triplicate.

The specific surface area (SSA) of the synthesized powder was analyzed using a nitrogen adsorption system Quadrasorb S1 (Quantachrome instruments, Boynton, FL, United States) by Brunauer-Emmett-Taylor (BET) method. Before analysis, the samples were degassed at room temperature for 24 h.

### 2.3 *In vitro* cytocompatibility

The preosteoblast (MC3T3-E1) cell line, obtained from ATCC, United States, was used in this study. Cells were grown in α-MEM (alpha-modified minimum essential medium eagle) with 10% fetal bovine serum (FBS) and 1% penicillin and streptomycin (pen-strep). The cells were cultured in 75 cm^2^ flasks and maintained under 5% CO_2_ at 37°C until the cell confluency reached 70%. The medium was replaced every 2 days.

For cellular analysis, suspensions were prepared by adding 10 %w/v ACP precipitate in α-MEM medium and incubated at 37°C in a humidified atmosphere of 95% air and 5% CO_2_ for 24 h. The extracts were collected by centrifugation at 350 rpm for 5 min and filtered to eliminate solid particles. The extracts were diluted with α-MEM medium to get the desired concentration of 1 %w/v. Therefore, the total sample concentration comprises 10 %w/v and 1 %w/v of each ACP. The extracts were then added to MC3T3-E1 cells containing well plates and incubated for 48 h. The α-MEM medium was added as a positive control, whereas the α-MEM medium with 6 %vol DMSO (dimethyl sulfoxide) was utilized as a negative control. Each sample was prepared in triplicate, and the same procedure was performed for all ACP variants.

Cell viability was measured by Cell counting kit-8 (CCK-8) (Sigma Aldrich, United States). Briefly, cells at a density of 1 × 10^4^ per well were seeded in a 96-well plate and pre-incubated at 37^ο^C under 5% CO_2_ for 24 h. After 24 h, the cell culture medium was replaced with various concentrations of ACP extracted medium [0 (as positive control), 10 %w/v, and 1 %w/v] and was further incubated for 48 h. CCK-8 was performed following the manufacturer’s protocol to determine cell viability. Cell viability (in percent) was determined as the absorbance ratio between cells grown in the presence and absence of extracted solutions. The average values and standard deviations were calculated from six replicate samples. 6 %v/v DMSO was used as a negative control. The experiments were performed in triplicate, and cytocompatibility was evaluated by calculating cell viability using Eq. 1:

$$Cell\;viability\;\left(\%\right)=\frac{\left(Absorbance\;of\;sample-Absorbance\;of\;blank\right)}{\left(Absorbance\;of\;positive\;control-\;Absorbance\;of\;blank\right)}\;x\;100$$ (1)

### 2.4 *In vitro* biomineralization

The *invitro* biomineralization experiments were performed with six types of ACP (pure ACP, ACP_ACE, ACP_GLU, ACP_ITN, ACP_ASC, and ACP_CIT). The ACP variants, such as pure ACP, ACP with citrate (ACP_CIT), and acetate (ACP_ACE), were synthesized, and characterization was reported in our previous study (Indurkar et al., 2023b). On the other hand, ACP variants such as ACP_GLU, ACP_ITN, and ACP_ASC were synthesized and characterized in the current study.

**FIGURE 2:**
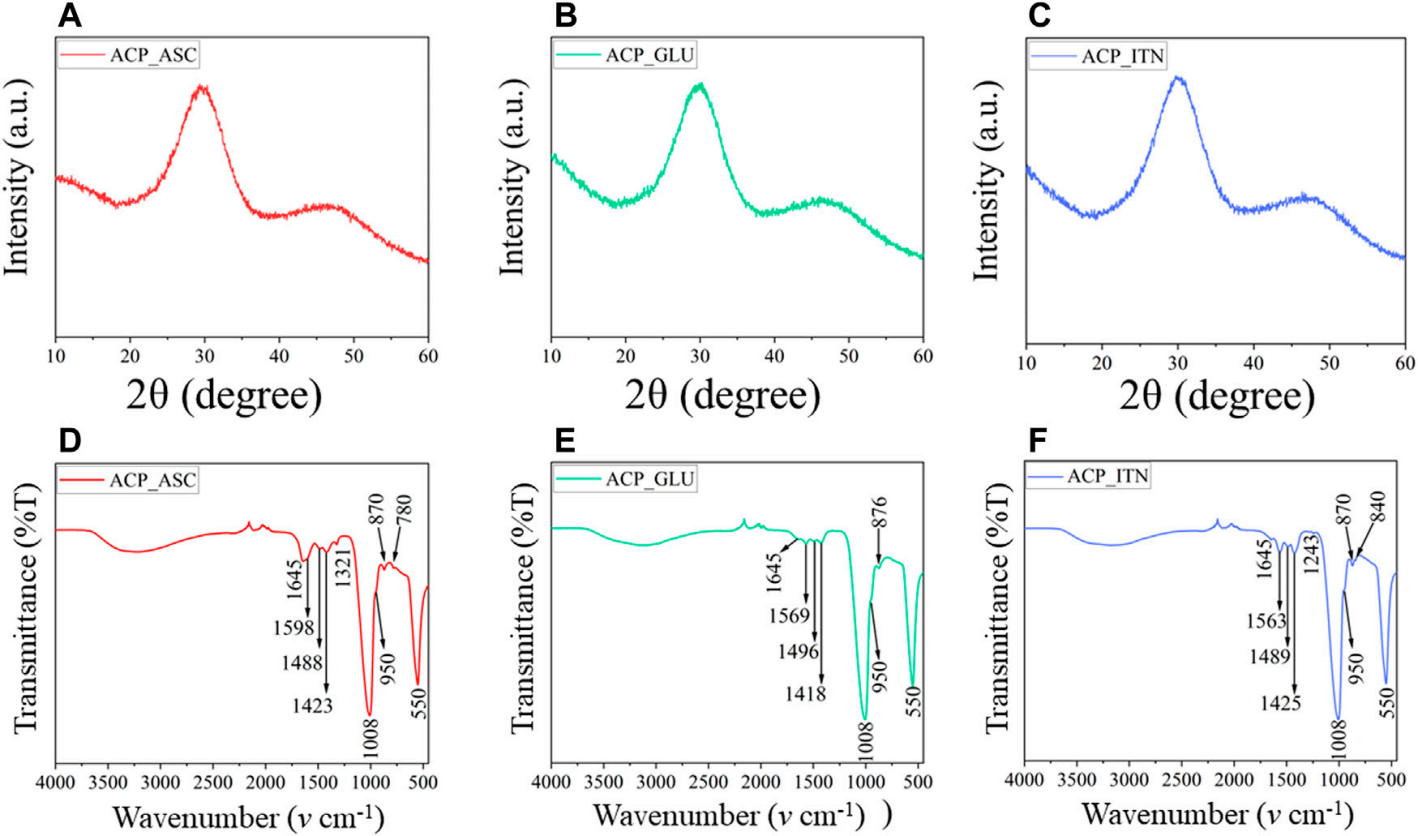
XRD **(A–C)** analysis confirms the amorphous nature of synthesized ACP with respective small compounds. The functional groups of ACP and the small compounds were revealed by FTIR **(D–F)** analysis.

Experiments were performed in batch mode. To analyze the effect of small organic molecules containing ACP on transformation to Ap, 100 mg of ACP was added into 5 mL of media (Milli-Q^®^ water, PBS, and α-MEM) preheated at 37°C. Further, the samples were incubated at 37°C and removed after specific time points (15, 30, 60, 120, 240, 1,440, 2,880, and 4,320 min) followed by centrifugation, freezing in liquid nitrogen for 15 min and freeze-drying for 72 h. The obtained powders were used for characterization as shown in Figure 2.

The obtained powders after freeze drying were analyzed using X-ray diffraction, performed with a Malvern Panalytical Aeris diffractometer (Netherlands). The diffraction data were collected at 40 kV and 15 mA in a step mode with a step size of 0.04°, in the 2θ range from 10° to 60°. This was followed by Fourier-transformed infrared spectroscopy (FTIR) analysis performed using a Nicolet iS50 FT-IR spectrometer (Thermo Scientific, Waltham, MA, United States). Experiments were performed in transmission mode in the range from 4,000 to 400 cm^−1^ with a resolution of 4 cm^−1^ (64 scans).

### 2.5 Statistical analysis

GraphPad Prism (GraphPad Software, San Diego, United States) was utilized to perform statistical analysis by two-way ANOVA and Tukey’s multiple comparisons. Probability (P) values *p < 0.05 **p < 0.01 were considered the statistically significant differences. The results were expressed in mean ± standard deviation (S.D.).

## 3 Results and discussion

### 3.1 Synthesis and characterization

The synthesis of ACP_ASC and ACP_ITN showed color changes during the reaction. For instance, a colorless solution was formed when ascorbic acid was added to calcium chloride. However, as the pH of the solution increased to 11.5 using 3 M NaOH, a light-yellow solution was obtained. Therefore, the resultant ACP_ASC powder was light-yellow. Similarly, the color change was observed during the synthesis of ACP_ITN; when the itaconic anhydride was added to the calcium chloride, a colorless solution was formed, which changes to light brown when pH was increased to 11.5 using 3 M NaOH. Therefore, the resultant ACP_ITN powder had a brownish tinge. No color change was observed during the synthesis of ACP_GLU; therefore, a white powder was obtained.

#### 3.1.1 XRD and FTIR analysis

The lack of crystalline order was observed in Figures 2A–C, confirming the formation of X-ray amorphous ACP. The FTIR spectra are shown in Figures 2D–F. The broad band of water observed between 3,000 cm^−1^ and 3,700 cm^−1^ corresponds to asymmetric and symmetric vibrations, and the band detected at 1,645 cm^−1^ represents the bending mode of water. These bands were observed in all the synthesized ACPs. In FTIR, the phosphate group shows four vibrational domains: *v1* (∼950 cm^−1^), *v2* (400–470 cm^−1^), *v3* (1,000–1,150 cm^−1^), and *v4* (500–620 cm^−1^). The phosphate vibrational peaks at *v1, v3,* and *v4* were observed in all the synthesized ACP variants (Indurkar et al., 2023a).

The ascorbate bands in ACP_ASC were observed at ∼3,000 cm^−1^ attributed to C-H vibrations. The bands between 1,500 cm^−1^ and 1,660 cm^−1^ indicate the presence of C=O and C-O vibrations (Williams and Rogers, 1937). The band at 1,488 cm^−1^ represents the CH bending, 1,423 cm^−1^ (CH_2_ scissoring), 1,321 cm^−1^ (CH bending), 871 cm^−1^ (C-C ring stretching), and 780 cm^−1^ (OH out of plane deformation) (Yohannan Panicker et al., 2006; Bichara et al., 2010; Dabbagh et al., 2014).

The glutamate bands in ACP_GLU were observed between 3,000 cm^−1^ and 3,700 cm^−1^, which also corresponds to -NH_2_ vibrations (Cárdenas-Triviño et al., 2020). The bands at 1,569 cm^−1^ and 1,418 cm^−1^ correspond to the asymmetric and symmetric vibration of C=O (Barth, 2000). The bands observed in 1,496 cm^−1^ were attributed to symmetric and asymmetric stretching of C-O of carboxylate ion (De Castro and Cassella, 2016). The band observed at 876 cm^−1^ represents C-C vibration of glutamate (Sumayya et al., 2008).

The itaconate bands in ACP_ITN were observed at 1,645 cm^−1^ and attributed to C=C vibrations. The band at 1,563 and 1,425 cm^−1^ corresponds to the asymmetric and symmetric vibration of C=O (Damilola Olawale et al., 2020). Moreover, the band observed at 1,489 cm^−1^ and 1,243 cm^−1^ corresponds to O-C-O and C-O stretching (Loginova et al., 2016; Wibowo et al., 2018). The band observed at 870 cm^−1^ and 840 cm^−1^ indicates CH stretching vibrations (Uhanov et al., 2020).

#### 3.1.2 NMR analysis

**FIGURE 3:**
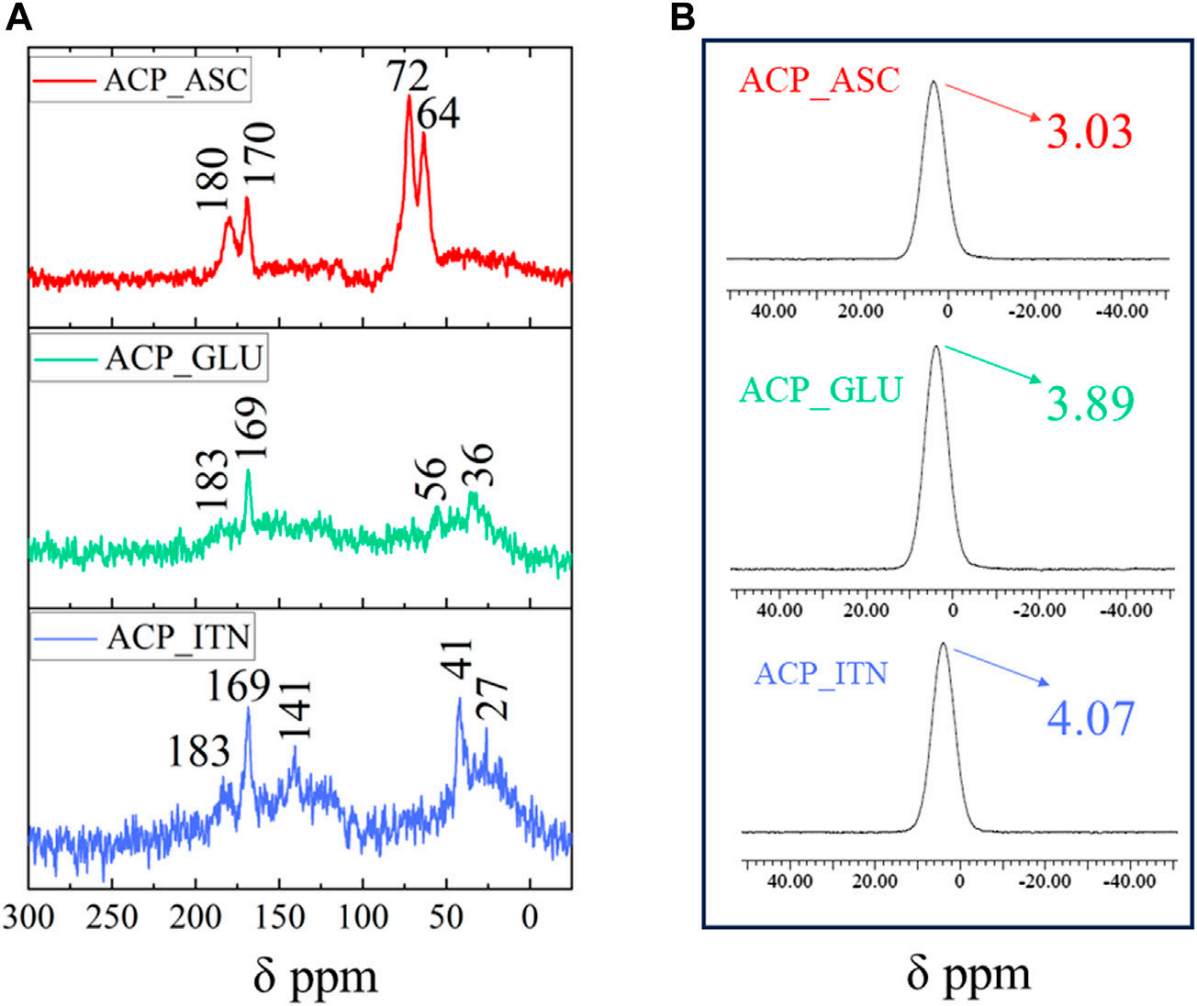
Solid-state NMR analysis of synthesized ACP with respective organic compounds **(A)** ^13^C NMR and **(B)** ^31^P NMR.

The solid-state ^13^C and ^31^P NMR analysis of synthesized ACPs is shown in Figures 3A, B. The ascorbate signals in ^13^C NMR analysis of ACP_ASC show the lactone ring and β-carbon carbonyl groups in the range of 170–180 ppm, corresponding to C=O of the carbonyl group. The carbon atoms at γ-,δ-, and ε-positions of ascorbate appeared at 72 and 64 ppm (Tajmir-Riahi, 1991). The interaction of ACP resulted in the shifted peaks of ascorbate.

Glutamate signals in ^13^C NMR analysis of ACP_GLU were observed at 183 and 169 ppm correspond to the carboxylate signals. The signal observed at 58 and 36 ppm was assigned to the carbon atom of the methine group bonded to the amino and carboxyl group (C2) and those branches in the alkyl chain (C3 and C4), respectively. The peak of the C4 carbon was shifted by the disordered conformation of the methylene group due to the association of ACP (Ikawa et al., 2009; Sannelli et al., 2023).

Itaconate signals in ^13^C NMR analysis of ACP_ITN were observed at 183 and 163 ppm, corresponding to the carboxyl group. The double bond signals were observed at 141 ppm. The carbon atom at the gamma position relative to the carboxyl group in the side chain appeared at 42 ppm, and the carbon atom of the methylene group was observed at 27 ppm (C3) (Strelko et al., 2011; Jahandideh et al., 2018; Vettori et al., 2022). The association with ACP resulted in peaks shifting in all the organic compounds.

**FIGURE 4:**
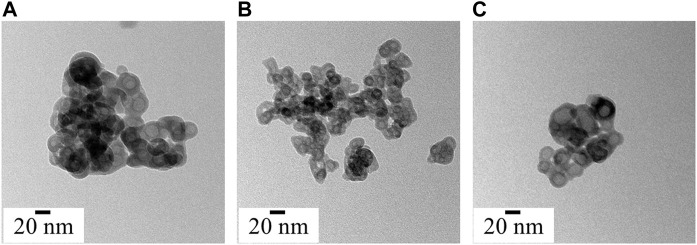
Morphology and particle size analysis of synthesized ACP variants **(A)** ACP_ASC, **(B)** ACP_GLU and **(C)** ACP_ITN.

The ^31^P NMR spectra of ACP show characteristic broad Gaussian peaks between −15 and 15 ppm centered from 2.2 to 6.5 ppm (Indurkar et al., 2023b). As shown in Figure 4B, the broad peak was observed in all the synthesized ACPs centered at 3.03 ppm (ASP_ASC), 3.89 ppm (ACP_GLU), and 4.07 ppm (ACP_ITN) respectively. The NMR analysis has confirmed the formation of ACP in the presence of the respective organic compounds (ascorbate, glutamate, and itaconate).

#### 3.1.3 Density and Brauner-Emmette-Teller (B.E.T.) analysis

The density and the specific surface area (SSA) of the synthesized ACP are shown in Table 1. The difference in density and SSA was observed, indicating the effect of the type of carboxyl ions on the synthesized ACP. One or several of the possible mechanisms can facilitate the interaction of ACP with organic compounds: 1) surface adsorption of organic compounds by chelating action of calcium ions of ACP and the carboxyl groups of organic compounds (Gorbunoff, 1984; Zong et al., 2023); 2) Organic compounds bearing two carboxylate ions (^−^O(O)C-R-C(O)O^−^) can substitute the hydrogen phosphate ion (HPO_4_^2−^) during the precipitation of ACP (Yokoi et al., 2022); 3) interaction between phosphates and carboxylates groups (P-O···H-O-C or P-O-H···O-C) (Corbridge, 1971). 4) Interaction of ascorbate anion can interact with calcium and/or phosphate group (Xu et al., 2019). However, more advanced analysis is required to evaluate the exact interaction of ACP with the organic compound.

**TABLE 1:** The density and specific surface area of the synthesized ACP.

Sample	Density (g/cm^3^)	SSA (m^2^/g)
ACP_ASC	2.82	115.2
ACP_GLU	2.64	92.4
ACP_ITN	2.43	130.3

#### 3.1.4 Morphological analysis

Under electron microscopy, the morphological appearance of ACP shows spherical particles of a few tenths of the nanometre scale (Zhao et al., 2012). All the synthesized ACPs show the characteristic spherical morphology, as shown in Figure 4. Numerous research groups have consistently identified the biomimetic size range of ACP, typically falling between 10 and 50 nm (Niu et al., 2014; Hoeher et al., 2023). Interestingly, the ascorbate, glutamate, and itaconate ACP variants show spherical hollow particles that align with the biomimetic size range. Moreover, ACP is highly sensitive to the electron beam and crystalizes on high electron beam exposure (Lotsari et al., 2018). The crystallization of synthesized ACP under a high electron beam is presented in Supplementary Figure S1.

#### 3.1.5 Cell culture analysis

**FIGURE 5:**
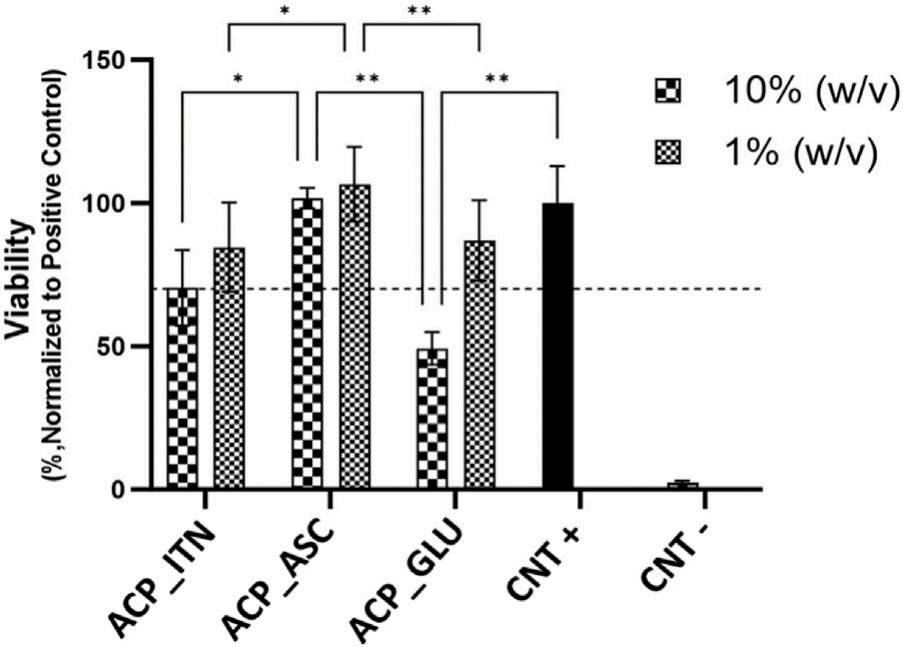
Relative cell viabilities of MC3T3-E1 cells cultured with extracts of ACP variants (10% and 1% w/v) prepared in α-MEM medium. All the samples were analyzed in triplicate, and data is presented in average and standard deviation. The CNT + and CNT–were positive and negative controls.

The cytocompatibility was evaluated by analyzing the cell viability of MC3T3-E1 cells in the presence of synthesized ACP extracts (10% and 1% w/v) prepared in α-MEM medium, as shown in Figure 5. The absorbance recorded for the cells cultured in the plain cell culture medium was normalized to 100% and termed positive control (CNT+). The cells cultured in 10% w/v ACP_GLU showed the lowest cell viability compared to other groups. The higher glutamate concentration can lead to excitotoxicity and or oxidative glutamate toxicity (Schubert and Piasecki, 2001; Kritis et al., 2015). However, reducing the concentration to 1% w/v, ACP_GLU enhanced the cell viability. The concentration of 10% w/v ACP_ITN and 10% w/v ACP_ASC was better than 10% w/v ACP_GLU. A similar trend was found in 1% w/v ACP_ITN and 1% w/v ACP_ASC. The cell viability of 10% and 1% w/v ACP_ASC was better than CNT+. Ascorbate is critical for the differentiation of the preosteoblast, and this may be the reason for the higher cell viability (Hadzir et al., 2014; Hwang and Horton, 2019). The preliminary analysis has confirmed the cytocompatibility of ACP_ASC (10% and 1% w/v), ACP_ITN (10% and 1% w/v), and ACP_GLU (1% w/v).

#### 3.1.6 *In vitro* biomineralization

The first *in vitro* biomineralization was investigated by Boskey and Posner by examining the impact of pH on the conversion of ACP to Ap. Their finding has provided critical insights revealing that transformation rate increases with higher pH levels. Furthermore, it was also discovered that the conversion pathways remain unaffected by the following factors: a) the nature of the buffer system used, b) the presence of a different type of univalent ions, and c) whether the material was in contact with mother liquor or filtered, dried, or added to the fresh buffer. Additionally, the transformation rate exhibited dependency on several factors, including smaller particle size, faster stirring rate, and higher ACP concentration (Boskey and Posner, 1973). Temperature also emerged as a significant variable affecting the conversion of ACP. For instance, at pH of 7.5, ACP converts five times more rapidly at 37°C than at 25°C. The synthesis condition of ACP was also found to influence the transformation rate (Eanes, 1998). Given the focus of our study on the application of ACP and its variants in tissue engineering, we consistently maintained 37°C in all the transformation experiments.

XRD analysis was employed to track alteration in long-range structural order as samples underwent successive progressive crystallization in different solvents, as shown in Supplementary Figure S2. In the analysis of CaP material, it is conventionally assumed that any part of the diffractogram not corresponding to the crystalline phase is amorphous. Nonetheless, there is ambiguity between non-coherent diffraction domains and genuinely separated amorphous phases. In the case of CaP (except hydroxyapatite), various ionic substitutions, defects, and vacancies can disrupt the regularity of the atomic array. This can significantly increase background diffraction patterns without necessarily implying the presence of any amorphous phase or domain (Combes and Rey, 2010). Therefore, FTIR analysis was also employed to provide additional insights. The FTIR spectra of all the ACP variants are shown in Supplementary Figure S3. The characteristic bands of ACP and the respective small organic molecules are shown in Supplementary Table S1).

**FIGURE 6:**
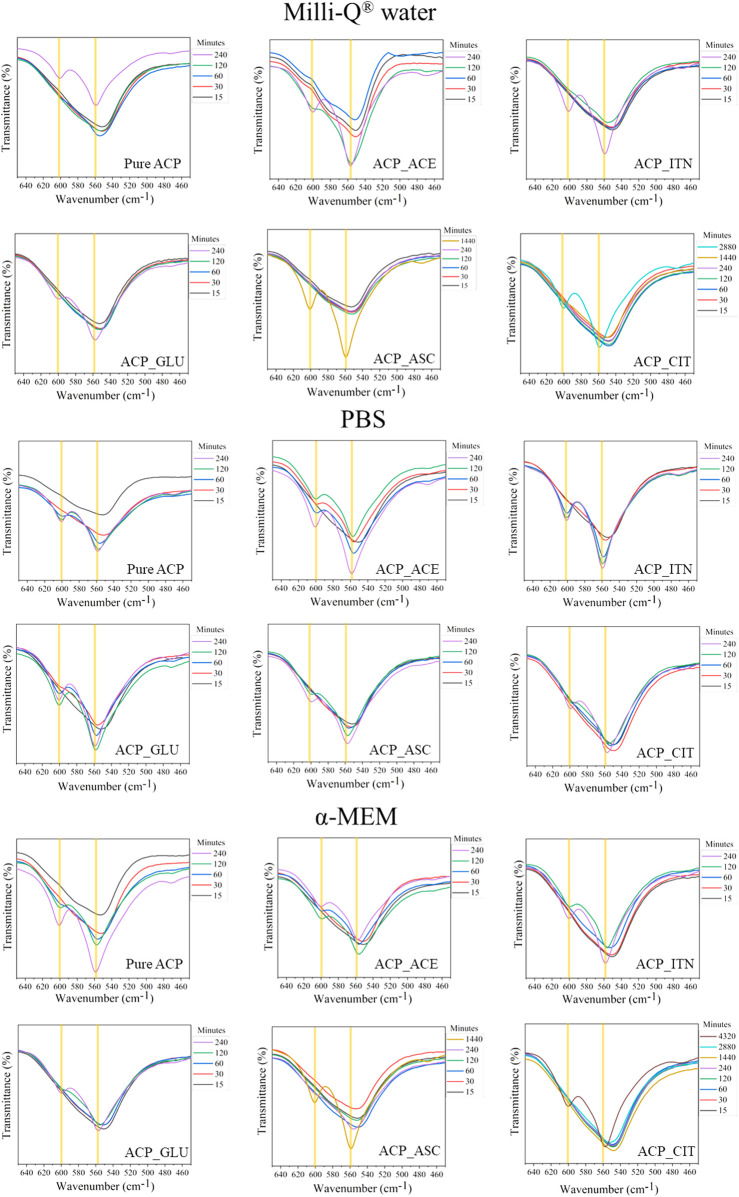
The stability of ACP in the different mediums was evaluated based on *v*_*4*_ PO_4_^3−^ vibration region. The samples that did not show evident splitting in the v_4_ PO_4_^3−^ vibration region was termed amorphous; on the contrary, the samples that showed splitting of v_4_ PO_4_^3−^ vibration region were termed crystalline.

FTIR spectral examination of ACP variants exhibits a distinct short order evident through absorbance band related to phosphate absorption bands. Notably, the ACP spectrum displays a broader band with no evident splitting in the *v*_*4*_ PO_4_^3−^ vibration region. The highlights distinction between XRD, which primarily detects specific peaks associated with crystalline phases, and FTIR, which detects absorbances originating from both amorphous and crystalline components (Querido et al., 2020). The splitting of the *v*_*4*_ PO_4_^3−^ vibration region confirms the crystallization of ACP, as shown in Figure 6. XRD analysis was utilized to complement the data obtained from FTIR analysis, and the stability time of the ACP variant in the respective medium is presented in Table 2.

**TABLE 2:** Time required for transformation of ACP to Ap evaluated from peak formation in XRD and splitting of the *v*_*4*_ PO_4_^3−^ vibration observed in FTIR spectra.

Samples	Milli-Q^®^ water (min)	PBS (min)	α-MEM (min)
Pure ACP	120	30	60
ACP_ACE	60	15	30
ACP_ITN	120	30	60
ACP_GLU	120	30	60
ACP_ASC	240	60	120
ACP_CIT	1,440	120	2,880

The inorganic contentment in the respective solvents is presented in Supplementary Table S2. The inorganic ions were absent in Milli-Q^®^ water; therefore, the transformation rates of ACP variants (except ACP_CIT) were slower compared to PBS and α-MEM medium. In PBS, all the synthesized ACP variants underwent a rapid transformation, aligning perfectly with findings from the literature (Zhao et al., 2012).

Previous studies have revealed that in the presence of PBS solution, the organic molecules are released from the surface of ACP, likely due to ionic exchange with the phosphate groups in the medium. This leads to an elevated concentration of phosphate in ACP, thus reducing stability and resulting in rapid transformation to low crystalline apatite (Chatzipanagis et al., 2016). This outcome was consistently observed for all the synthesized ACP variants. The phosphate content in α-MEM medium was less compared to PBS; therefore, the transformation rates were slower (except ACP_CIT). In the case of ACP_CIT, the highest stability of 2,880 min was observed in α-MEM medium. Previous studies have shown the interaction of serum albumin with citrate-stabilized gold nanoparticles forming protein corona (Dominguez-Medina et al., 2012; Zhang et al., 2014; Szekeres and Kneipp, 2018). Similarly, the delayed transformation of ACP_CIT may be due to the interaction of negatively charged citrate with FBS present in α-MEM medium. However, in-depth analysis is required to confirm this phenomenon.

**FIGURE 7:**
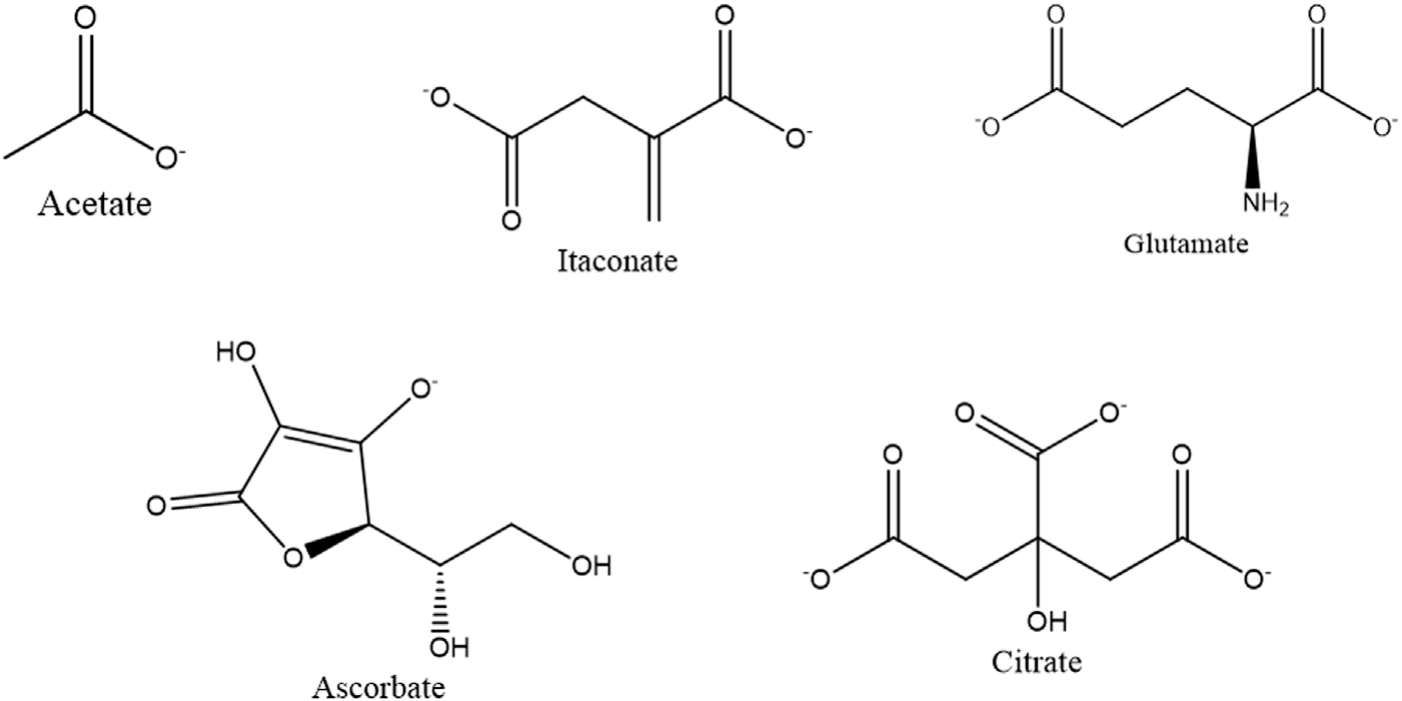
Small organic compounds are utilized for the synthesis of ACP variants.

The organic molecules such as acetate, itaconate, glutamate, and citrate contain carboxyl groups, as shown in Figure 7. Both the carboxyl and ascorbate anion have the potential to interact with both calcium and phosphate ions present in ACP (Winand et al., 1961; Corbridge, 1971; Ancillotti et al., 1977; Chatzipanagis et al., 2016). Due to the combination of these factors, the interaction between these organic compounds and ACP is complex and demands a sophisticated analysis to fully comprehend the precise nature of these interactions.

**FIGURE 8:**
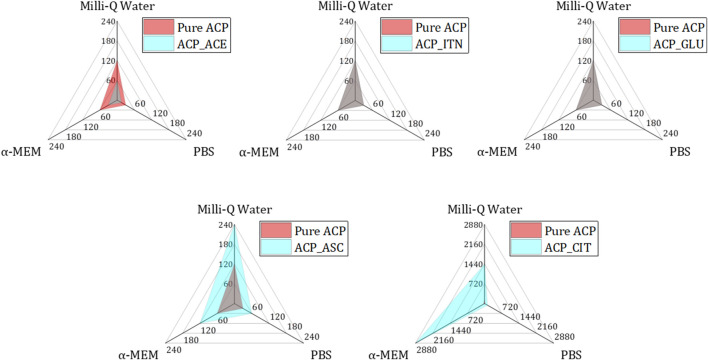
Analysing the transformation rates of small organic molecules containing ACP (blue) in comparison to pure ACP (red) across different mediums (Milli-Q^®^ water, PBS, and α-MEM) reveals distinct patterns. Specifically, ACP_ACE exhibited accelerated transformation rates in all mediums compared to pure ACP (both represented in grey colour by combination of blue and red). Conversely, ACP_ITN and ACP_GLU demonstrated similar transformation rates to pure ACP. Notably, ACP_GLU and ACP_CIT displayed slower transformation rates compared to their pure ACP counterpart.

To investigate the effect of small organic molecules on transformation rate, pure ACP was used for comparison as shown in Figure 8. The variations in the transformation rate can be attributed to the changes in the physiochemical properties of ACP, such as particle size, morphology, specific surface area (SSA), and density induced by different functional groups from the respective small organic molecules, as illustrated in Supplementary Table S3.

The fastest conversion rate was observed in ACP_ACE in all the respective mediums compared to other ACP variants. Acetate consists of one carboxyl group (COO^−^) representing a negative charge of −1. Monocarboxylic ions can interact with calcium ions but are not capable of bridging two calcium ions. The incompatible charge of −1 promotes the formation of HAp (Yamada et al., 2021). A similar phenomenon is observed in the case of fluoride-doped ACP. Fluoride ions also have a negative charge of −1; therefore, the transformation of ACP to Ap was faster in the presence of fluoride-doped ACP than in pure ACP (Ten Cate and Featherstone, 1991; Iafisco et al., 2018).

Pure ACP, ACP_GLU, and ACP_ITN represent similar conversion rates in respective medium. Our previous study observed presence of carbonate (CO_3_^2-^) in pure ACP. The presence of carbonate ions in ACP is known to retard its conversion to Ap (Combes and Rey, 2010; Edén, 2021). Similarly, itaconate and glutamate are dicarboxylic compounds with a negative charge of −2 and may behave similarly to carbonate-substituted ACP. Therefore, the transformation rate of pure ACP, ACP_ITN and ACP_GLU was the same.

In the case of ascorbate containing ACP, the conversion was slower than that of other ACP variants (except ACP_CIT). The presence of the ascorbate anion can interact with ACP, thus retarding its conversion to Ap. Previous studies have shown delayed struvite crystallization in the presence of ascorbate due to a decrease in pH (Manzoor et al., 2018). Additionally, ascorbate in the bloodstream has been shown to prevent struvite stone formation in urine (Flannigan et al., 2014). Similarly, delayed sugar crystallization was observed in the presence of ascorbic acid (Zhou and Roos, 2012). Likewise, a delayed transformation was observed in ACP_ASC despite its higher surface area (115.2 m^2^/g). However, a more comprehensive analysis is required to confirm the exact behavior.

The highest stability was observed in ACP_CIT. Citrate is a tricarboxylic acid that can interact in four ways with ACP: a). The carboxyl group can interact with calcium ions of ACP. B) HCit^3-^ can substitute PO_4_^3−^ of ACP. c) The hydroxy group of citrates can interact with phosphate ions and/or d) The phosphate ions can interact with the carboxyl group of citrates (Indurkar et al., 2023b). Previous reports have also shown citrate’s efficacy in stabilizing ACP (Chen et al., 2014; Sakhno et al., 2023).

The ACP variants displayed a generally spherical and hollow morphology, except for ACP citrate, which exhibited a dense spherical structure. Differences in the morphologies may also contribute to the transformation rate. However, more detailed analysis is required to confirm this phenomenon.

## 4 Discussion

The synthesis reactions of ACP are known for their rapid nature and are typically performed under alkaline conditions. However, owing to the triprotic nature of phosphate ions, fluctuations in pH, temperature, and precursor concentration play a crucial role in determining the efficiency of precipitation and particle size of the synthesized product (ACP) (Mekmene et al., 2009).

**FIGURE 9:**
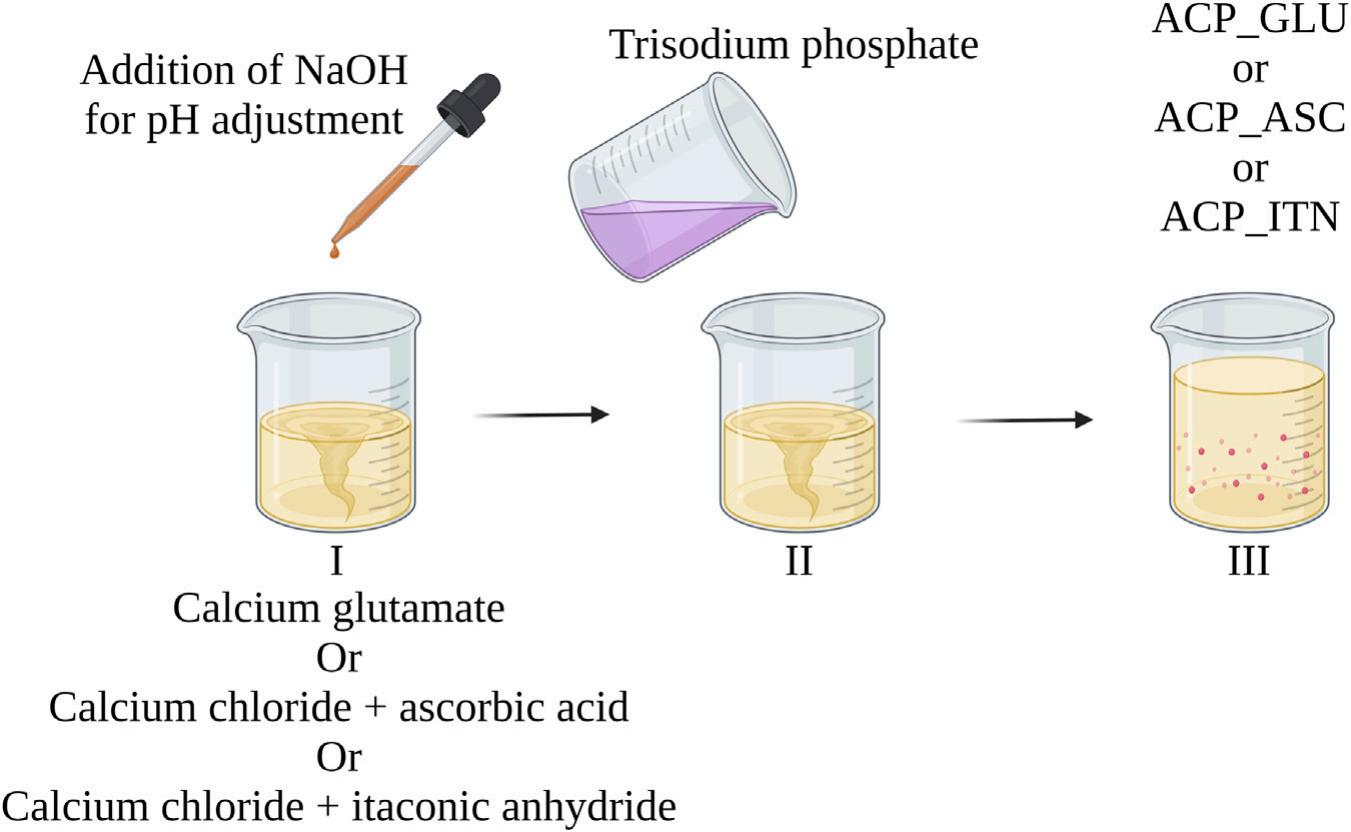
Illustrating the systematic approach to achieve controlled ACP (with organic compounds) synthesis with minimal pH variations. The approach was previously reported in developing pure ACP and ACP with citrate and acetate (Indurkar et al., 2023b).

To address these inherent challenges, we have devised a straightforward and effective method to synthesize ACP, aiming to minimize pH variations. Based on this approach, we have previously achieved successful ACP synthesis with citrate and acetate (Indurkar et al., 2023b). Inspired by this successful strategy, we have extended our efforts to synthesize ACP with other organic compounds such as ascorbate, glutamate, and itaconate. The schematic representation of our synthesis method is outlined in Figure 9. These methods have broadened the scope of ACP synthesized by incorporating diverse organic compounds, thus showcasing its versatility and potential for further exploration in developing novel ACP composites.

Our prior study also synthesized two pristine ACPs devoid of organic compounds (Indurkar et al., 2023b). The ACP particles exhibited an SSA of 105 m^2^/g and particle size of less than 20 nm. In that investigation, acetate and citrate have shown distinct effects on the properties of ACP. The surface area and particle size were affected when ACP was linked with acetate and citrate. Building on these findings, our current study investigated the effect of other organic compounds, ascorbate, glutamate, and itaconate, on the properties of ACP. Interestingly, introducing these compounds impacted the SSA and particle size. Moreover, an effect on cytocompatibility was also observed, wherein the 10% w/v ACP_GLU represented a toxic effect on MC3T3-E1 cells.

Glutamate is a versatile molecule with a potential role in influencing cellular processes and viability in bone tissue (Skerry and Taylor, 2005). It has been suggested that glutamate can impact cell proliferation and differentiation through group III metabotropic glutamate receptors expressed in mouse calvaria osteoblasts. Furthermore, previous investigations have revealed that specific concentrations within 50 μM to 1 mM enhance survival rates of human primary osteoblasts when exposed to tumor necrosis factor-α and interferon-γ. However, it is essential to note that a cytotoxic effect was observed when glutamate concentration exceeded 10 mM (Genever and Skerry, 2001).

The cytotoxicity resulting from elevated glutamate levels is attributed to the induction of oxidative stress. This occurs because of triggering the release of reactive oxygen species and nitric oxide. Consequently, excess glutamate concentration leads to oxidative damage to MC3T3-E1 cells, resulting in apoptosis (Fatokun et al., 2006). A similar behavior may be responsible for 10% w/v ACP_GLU samples resulting in cell death. However, further advanced analysis and experiments are required to confirm this phenomenon and gain a deeper understanding of the underlying mechanism.

Examination into *in vitro* biomineralization has revealed a significant influence of small organic molecules on the transformation rate of ACP. By aligning ACP with small organic molecules and solvents, transformation necessities can be tuned based on the applications. FTIR analysis has confirmed that the resulting Ap retained characteristics of the respective small organic molecules. This observation suggests the potential utilization of transformed Ap as an advanced bone substitute material.

## 5 Conclusion

The study introduces a promising avenue for biomimetic ACP developed by leveraging mitochondrial (ascorbate, glutamate, and itaconate) small organic molecules. Synthesized ACP variants were characterized using XRD, FTIR, and NMR, which confirmed the presence of respective organic compounds and the amorphous nature of ACP. Advanced analysis is required to understand the exact interaction of the organic compounds with ACP. *In vitro* analysis using MC3T3-E1 cells has provided primary evidence of cytocompatibility of compositions obtained. *In vitro* biomineralization studies have shown differences in the transformation rate of small organic molecules containing ACP. The variations in the transformation rate can be induced by different functional groups from the respective small organic molecules. The research contributes to the expanding field of biomaterial science by bridging the gap between the biomineralization process and synthetic material, thus opening the door to innovative tissue engineering strategies and bone therapeutic interventions.

## Data Availability

The raw data supporting the conclusion of this article will be made available by the authors, without undue reservation.
