# Supplementary material for: Small organic molecules containing amorphous calcium phosphate: synthesis, characterization and transformation
Source: Front Bioeng Biotechnol. 2024 Jan 12;11:1329752. doi: 10.3389/fbioe.2023.1329752 (PMC10811600; doi:10.3389/fbioe.2023.1329752)
Supplement: Supplementary file 1 [file DataSheet1.docx]

**Small Organic Molecules Containing Amorphous Calcium Phosphate: Synthesis, Characterization and Transformation**

Abhishek Indurkar,^1,2^ Pawan Kudale,^3^ Vitālijs Rjabovs,^4^ Ivo Heinmaa,^5^ Öznur Demir,^1,2^ Matvejs Kirejevs,^1^ Kristaps Rubenis,^1,2^ Ganesh Chaturbhuj,^3^ Māris Turks,^4^ and Janis Locs^1,2^*

^1^Rudolfs Cimdins Riga Biomaterials Innovations and Development Centre of RTU, Institute of General Chemical Engineering, Faculty of Materials Science and Applied Chemistry, Riga Technical University, Pulka Street 3, LV-1007 Riga, Latvia.

^2^Baltic Biomaterials Centre of Excellence, Headquarters at Riga Technical University, Kipsalas Street 6A, LV-1048 Riga, Latvia.

^3^Department of Pharmaceutical Sciences and Technology, Institute of Chemical Technology, Matunga, Mumbai, 400019, India.

^4^Institute of Technology of Organic Chemistry, Faculty of Materials Science and Applied Chemistry, Riga Technical University, P. Valdena 3, LV-1048, Riga, Latvia.

^5^National Institute of Chemical Physics and Biophysics, Akadeemia tee 23, 12618 Tallinn, Estonia.

*Corresponding author – Janis.Locs@rtu.lv

**Supplementary data**


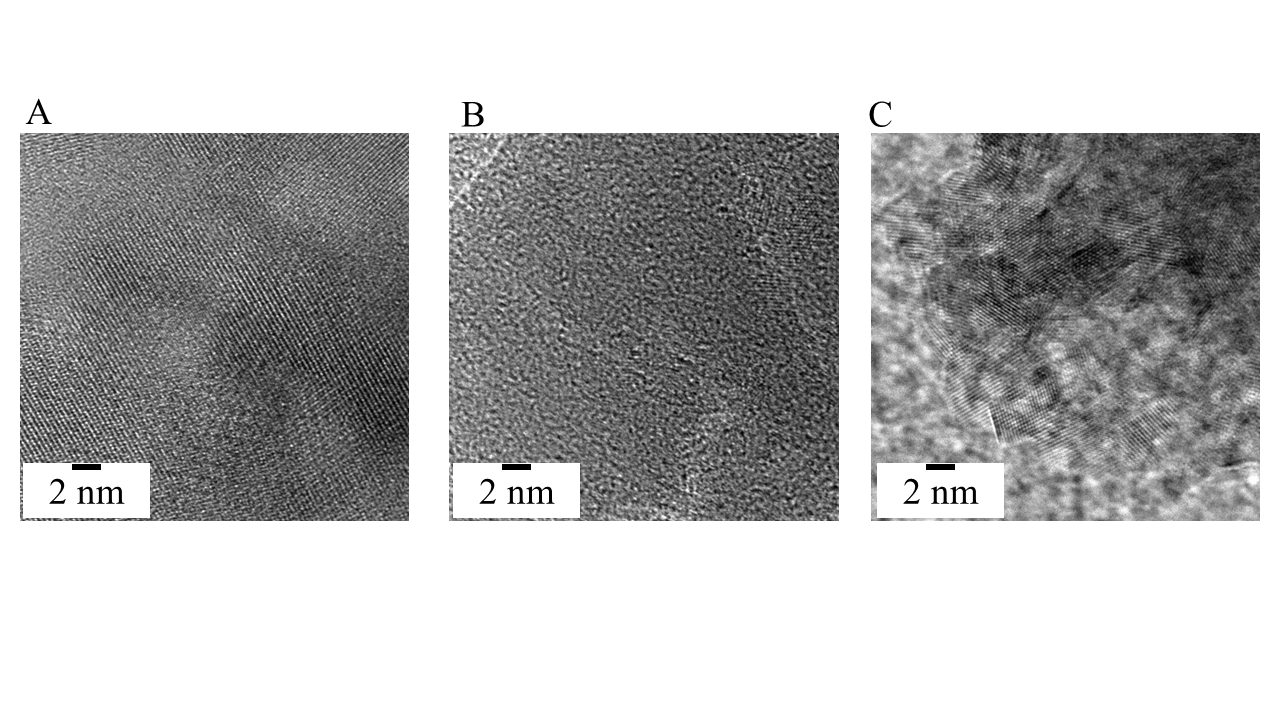


Figure S1. Crystallization of ACP variants under high electron beam A) ACP_ASC, B) ACP_GLU and C) ACP_ITN


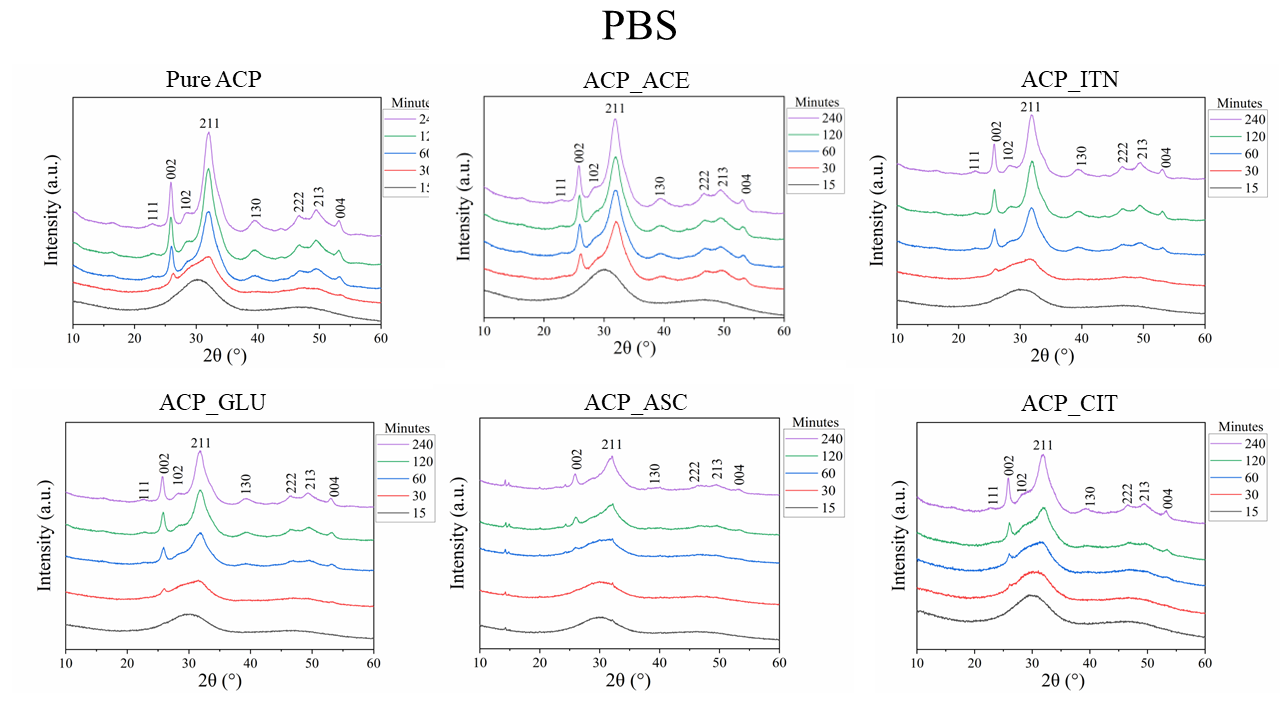

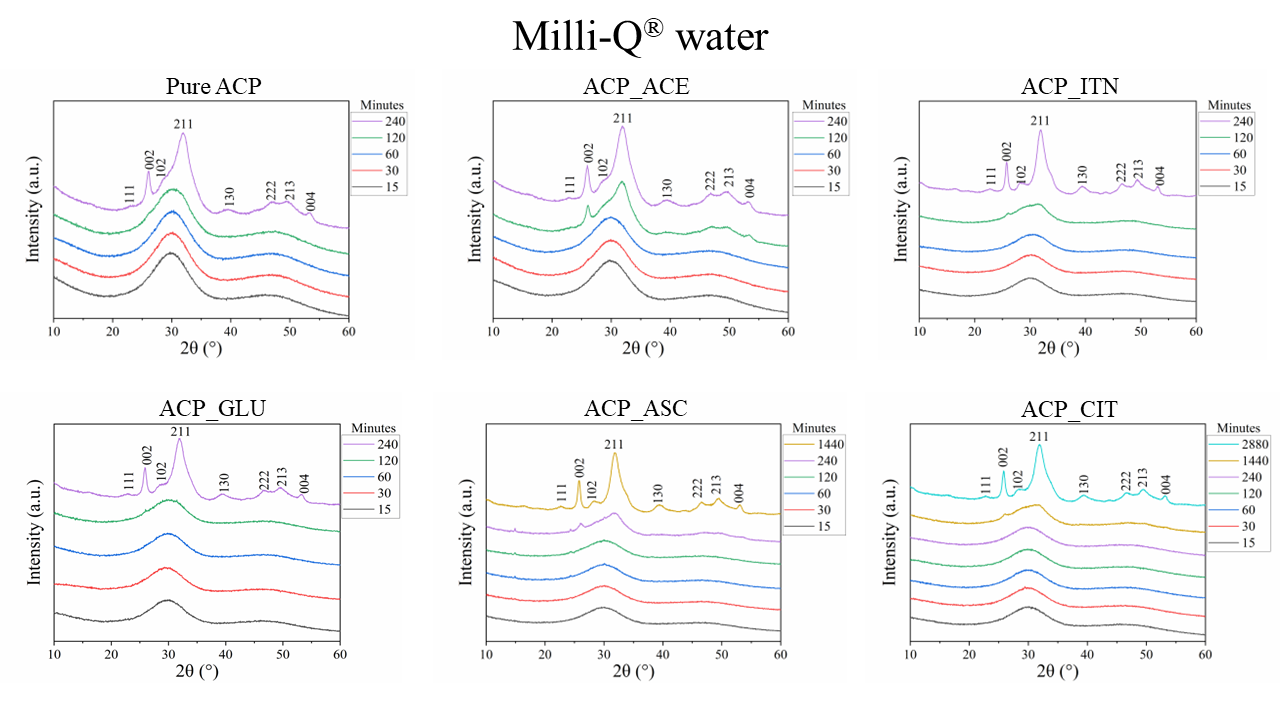


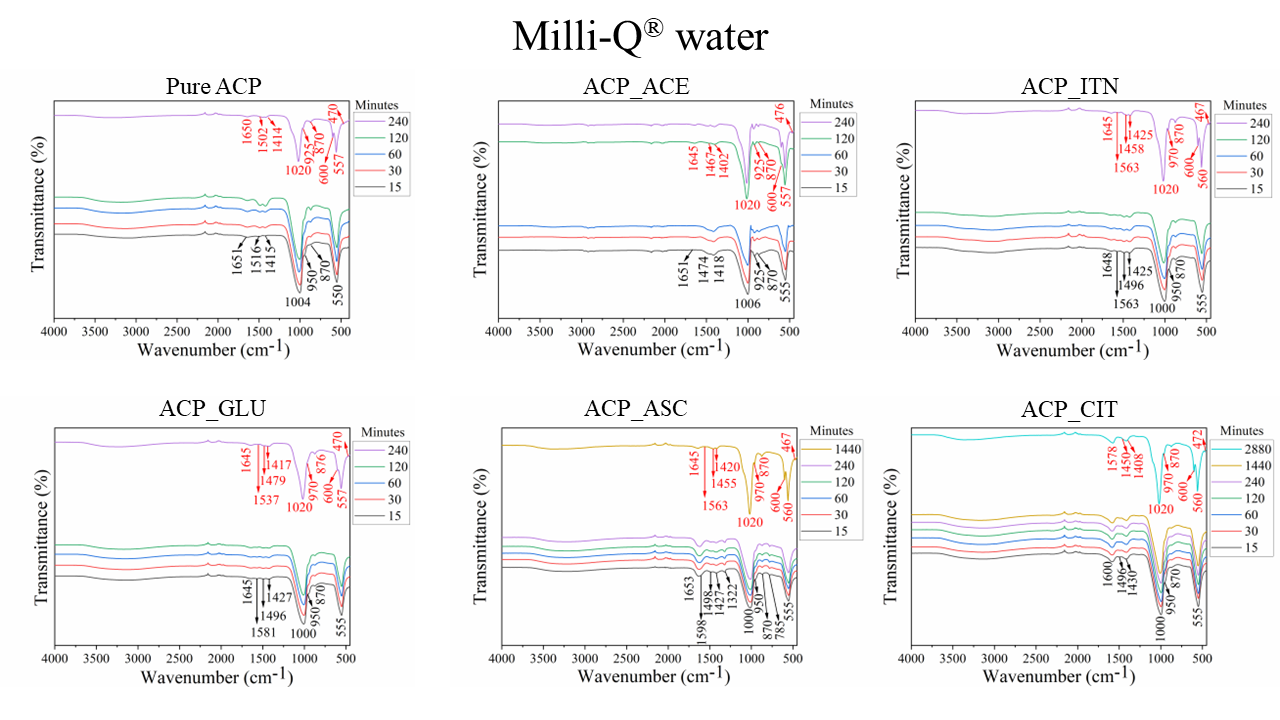

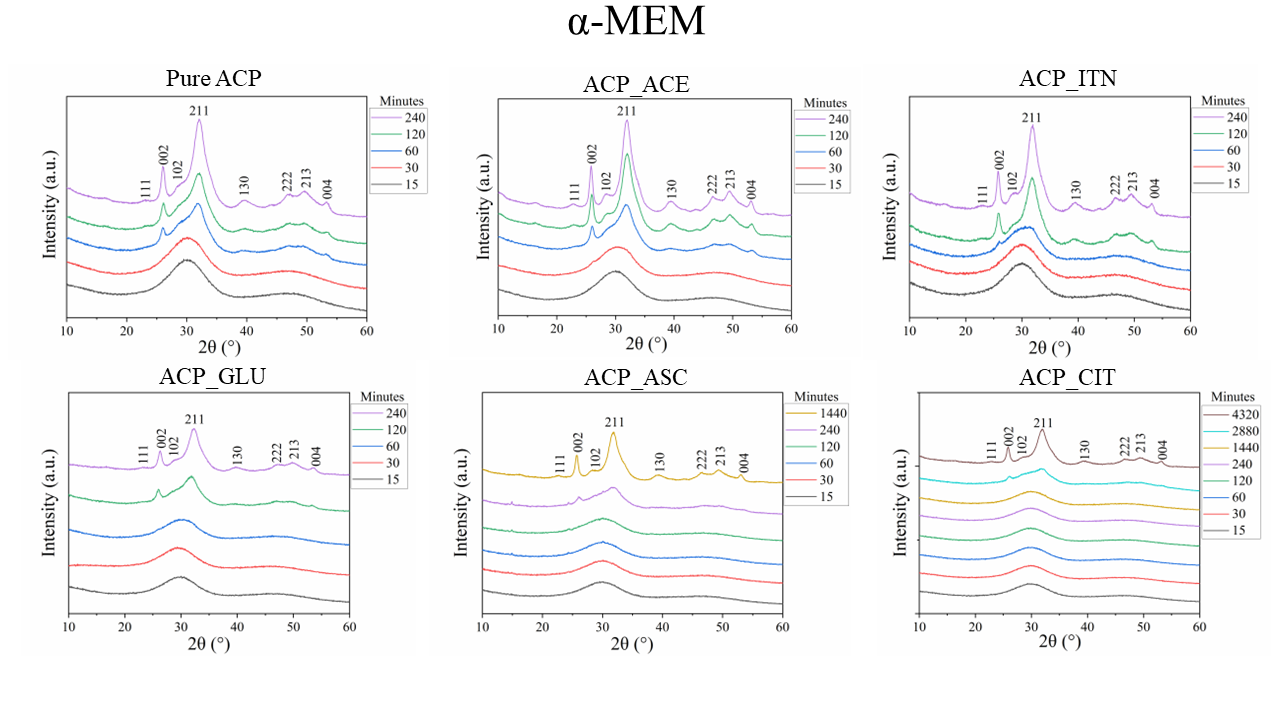
Figure S2. XRD analysis to track alteration and successive progression in crystallization of ACP in different solvents such as Milli-Q^®^ water, PBS, and α-MEM medium. The peaks were matched with standard ICDD card no. 00-064-0738.


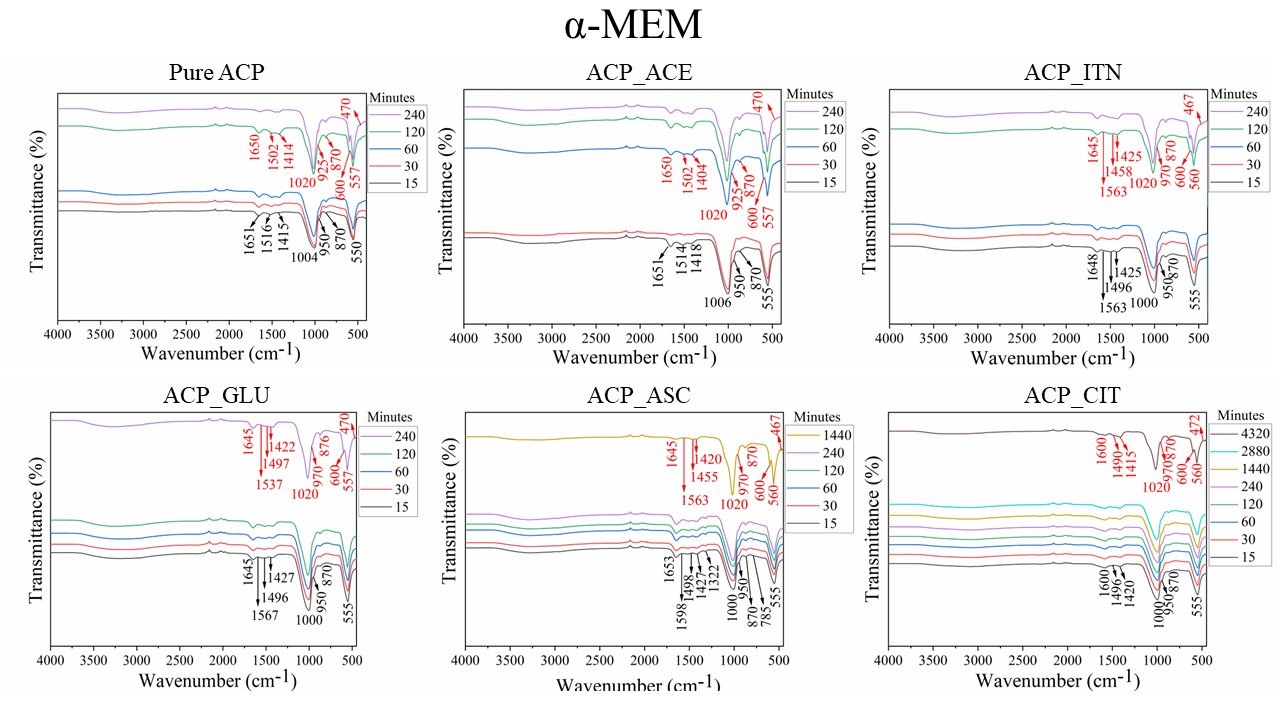

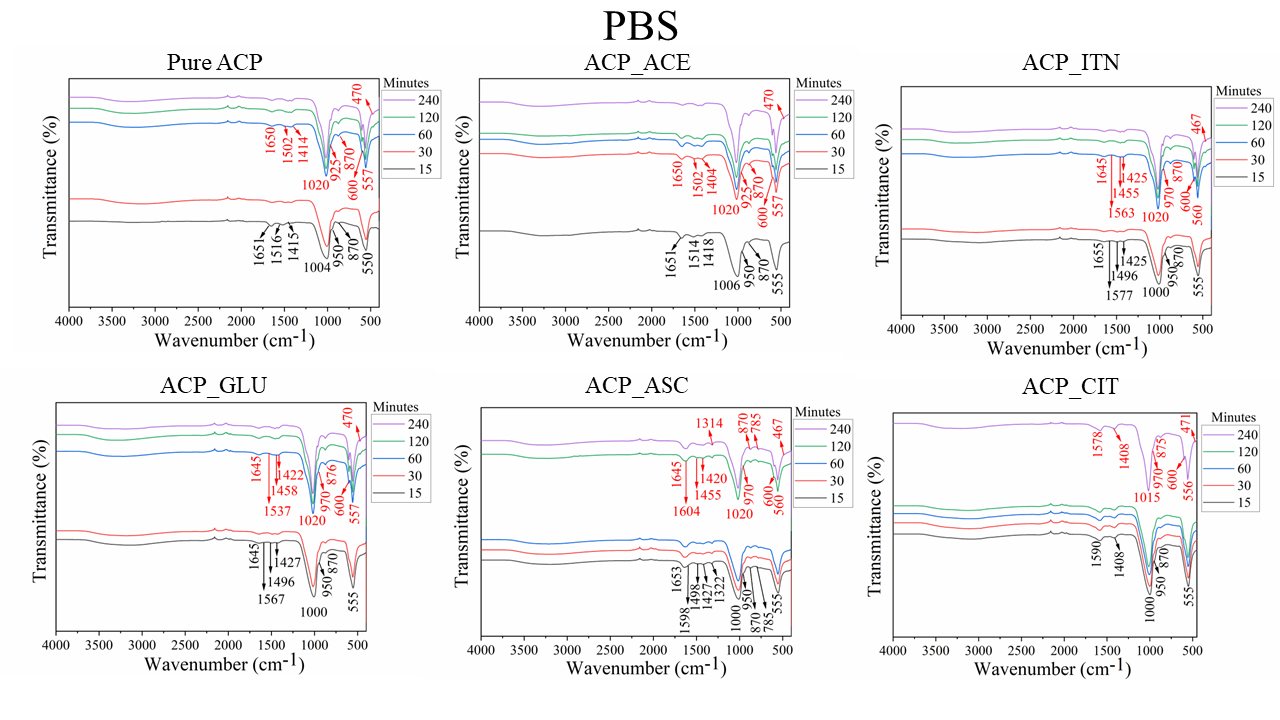


Figure S3. FTIR analysis to track alteration and successive progression in crystallization of ACP in different solvents such as Milli-Q^®^ water, PBS, and α-MEM medium.

**Table S1 – FTIR bands and corresponding functional groups of ACP and its variants.**

| **Sample** | **Bands (cm ^-1^)** | **Functional groups** | **Reference** |
| --- | --- | --- | --- |
| Pure ACP_CL | 3000 - 3700  1680 - 1640  1400 - 1550  1000 - 1150  950  875  500 - 620 | OH asymmetric and symmetric stretching mode  OH bending mode  *v_3_*CO_3_^2-^  *v_3_*PO_4_^3-^  *v_1_*PO_4_^3-^  *v_2_*CO_3_^2-^  *v_4_*PO_4_^3-^ | (Indurkar et al., 2023) |
| ACP_ACE | 3000 - 3700  1680 - 1640  1550  1440  1000 - 1150  950  600 - 680  500 - 620 | OH asymmetric and symmetric stretching mode  OH bending mode  COO^-^ bending  COH stretching.  *v_3_*PO_4_^3-^  *v_1_*PO_4_^3-^  COO^-^ bending  *v_4_*PO_4_^3-^ | (Indurkar et al., 2023) |
| ACP_ITN | 3000 - 3700  1680 - 1640  1563  1489  1425  1243  1000 - 1150  950  870 and 840  500 - 620 | OH asymmetric and symmetric stretching mode  OH bending mode and/or C=C vibrations  asymmetric C=O vibration  O-C-O stretching  symmetric C=O vibration  C-O stretching  *v_3_*PO_4_^3-^  *v_1_*PO_4_^3-^  CH stretching vibrations  *v_4_*PO_4_^3-^ | Reported in the current study |
| ACP_GLU | 3000 - 3700  1680 - 1640  1569  1496  1418  1117 - 870  1000 - 1150  950  500 - 620 | NH_2_ vibrations and OH asymmetric and symmetric stretching mode  OH bending mode  asymmetric C=O vibration  symmetric and asymmetric stretching of C-O  symmetric C=O vibration  C-C bending mode  *v_3_*PO_4_^3-^  *v_1_*PO_4_^3-^  *v_4_*PO_4_^3-^ | Reported in the current study |
| ACP_ASC | 3000 - 3700  ~3000  1500 - 1660  1488  1423  1321  1000 - 1150  950  871  780  500 - 620 | OH asymmetric and symmetric stretching mode  C-H vibrations  C=O and C-O vibrations of carboxylate ions  CH bending  CH_2_ scissoring  CH bending  *v_3_*PO_4_^3-^  *v_1_*PO_4_^3-^  C-C ring stretching  OH out of plane deformation  *v_4_*PO_4_^3-^ | Reported in the current study |
| ACP_CIT | 3000 - 3700  1680 - 1640  1600  1446  1000 - 1150  950  500 - 620 | OH asymmetric and symmetric stretching mode  OH bending mode  COO^-^ bending  COH stretching  *v_3_*PO_4_^3-^  *v_1_*PO_4_^3-^  *v_4_*PO_4_^3-^ | (Indurkar et al., 2023) |

**Table S2 – Inorganic compounds present in the respective mediums**

| Solvents | Milli-Q^®^ water | PBS  pH – 7.4 | α-MEM  pH – 7.4 |
| --- | --- | --- | --- |
| Sodium chloride | - | 8 g | 6.8 g |
| Potassium chloride | - | 0.2 g | 0.4 g |
| Sodium phosphate | - | 1.44 g | 0.014 g |
| Potassium phosphate monobasic | - | 0.245 g | - |
| Calcium chloride | - | - | 0.2 g |
| Magnesium chloride | - | - | - |
| Magnesium sulphate | - | - | 0.097 g |
| Sodium bicarbonate | - | - | 2.2 g |
| Dextrose | - | - | 1 g |
| Dipotassium phosphate | - | - | - |
| 1M HCl | - | - | - |
| Sodium sulphate | - | - | - |
| Tris(hydroxymethyl)aminomethane | - | - | - |

**Table S3 – Physiochemical properties of synthesised ACP and its composites**

| **Sample** | **Density (g/cm^3^)** | **BET (m^2^/g)** | **Morphology** |
| --- | --- | --- | --- |
| Pure ACP_CL | 2.62 | 105 | Spherical hollow (Indurkar et al., 2023) |
| ACP_ACE | 2.47 | 118 | Spherical hollow (Indurkar et al., 2023) |
| ACP_ITN | 2.43 | 130.3 | Spherical hollow (current study) |
| ACP_GLU | 2.64 | 92.4 | Spherical hollow (current study) |
| ACP_ASC | 2.82 | 115.2 | Spherical hollow (current study) |
| ACP_CIT | 2.57 | 62 | Spherical Dense (Indurkar et al., 2023) |

**References**

Indurkar, A., Choudhary, R., Rubenis, K., Nimbalkar, M., Sarakovskis, A., R. Boccaccini, A., et al. (2023). Amorphous Calcium Phosphate and Amorphous Calcium Phosphate Carboxylate: Synthesis and Characterization. *ACS Omega* 8, 26782–26792. doi: 10.1021/acsomega.3c00796.
